# Children’s mental health needs and access to specialized services in Mexico

**DOI:** 10.1192/j.eurpsy.2021.259

**Published:** 2021-08-13

**Authors:** L. Díaz-Castro, M. Márquez-Caraveo, H. Cornú-Rojas, M. Martínez Jaimes, M. García-Andrade, H. Cabello-Rangel

**Affiliations:** 1 Direction Of Epidemiological And Psychosocial Research, National Institute of Psychiatry Ramon de la Fuente Muñiz, Ciudad de México, Mexico; 2 Research Division, Child Psychiatric Hospital “Dr. Juan N. Navarro”, Ciudad de México, Mexico; 3 Division Of Medical Care, Psychiatric Hospital “Fray Bernardino Álvarez”, Ciudad de México, Mexico

**Keywords:** Children, Help-seeking, Mental disorders, Mental health-care services

## Abstract

**Introduction:**

The prevalence of mental disorders (MD) is greater in children; however, they are the population with less help-seeking and access to mental health-care services (MHS).

**Objectives:**

To explore the characteristics of help-seeking and access to specialized MHS in children with MD.

**Methods:**

A cross-sectional study was carried out from 2018 to 2019, in the Children’s Psychiatric Hospital and National Institute of Psychiatry in Mexico City. Sample 397 children and 397 caregivers. The project was approved by the Ethics Committee of both institutions. The patient’s family member was questioned on sociodemographic data and help-seeking to MHS. Sample’s descriptive statistics applying measures of central tendency, Inferential statistics with t-test for differences in means between groups (diagnosis), and one-way ANOVA to variables associated with the help-seeking to MHS.

**Results:**

Children´s sample: 37% female, average age 12 years (SD± 3.6), 51% had diagnosis of hyperkinetic disorder (HD), 34% depressive disorder (DD). The children´s age at the time of seeking healthcare was different according to the diagnosis: DD 10.1 (SD ± 4.5) and HD 6.95 (SD ± 3.4), (T = -3.18, p = 0.000); and by sex: girls 10.9 (SD ± 4.5), boys 7.85 (SD ± 4.0); (T = -3.07, p = 0.000). The mother was the first person to notice the symptoms.

**Conclusions:**

The search for MHS differs by sex, diagnosis and family history; it is necessary to design mental health interventions considering gender-based differences, namely, to integrate a gender perspective.

**Disclosure:**

No significant relationships.

